# Low-dose cyclosporine treatment for sight-threatening uveitis: Efficacy, toxicity, and tolerance

**DOI:** 10.4103/0301-4738.58472

**Published:** 2010

**Authors:** D Mathews, John Mathews, N P Jones

**Affiliations:** Uveitis clinic, Royal Eye Hospital, Manchester, UK

**Keywords:** Cyclosporine, immunosuppression, steroid, uveitis

## Abstract

**Aim:**

To ascertain the effectiveness, tolerability, and safety of low-dose cyclosporine in the management of sight-threatening uveitis.

**Materials and Methods:**

This was a retrospective clinical case series of patients using oral low-dose cyclosporine for the management of sight-threatening uveitis in the uvea clinic (UC). Patients receiving cyclosporine were identified from the clinic database. Main outcome measures were degree of intraocular inflammation, visual acuity and dose reduction of oral steroid for effectiveness and adverse symptoms, systemic hypertension, and raised serum creatinine for tolerability and safety.

**Results:**

Intraocular inflammation was improved or stable in 97% of patients, visual acuity was improved or stable in 91%, and oral steroid dosage was reduced in 73% (by half or more in 51%). Adverse symptoms were almost universal, the commonest being peripheral paresthesia/burning in 70% and fatigue in 67%. Significant systemic hypertension developed in 27% and raised creatinine in 30%, necessitating dose reduction. Cyclosporine was discontinued in 35%, being intolerable in 20% and ineffective in 15%.

**Conclusions:**

Cyclosporine was found to be effective in reducing inflammation and protecting vision in sight-threatening uveitis. It was safe with proper monitoring, including in children. It had a significant toxicity profile and a high incidence of adverse symptoms which required close supervision, and a prompt dose reduction or drug exchange.

Cyclosporine is a natural antibiotic discovered in 1972 by Borel *et al*.[1] and was found to have immunosuppressive properties in 1976.[2] It was first used as a clinical immunosuppressive in 1978 in renal transplant patients and its first ophthalmic use was to prevent the rejection of rabbit corneal transplant.[3] It was discovered to be effective in the treatment of experimental autoimmune uveitis in an animal model by Nussenblatt *et al*.[4] who also went on to report results for a small group of patients with uveitis in 1983.[5] Although the drug became more widely used for such patients including juveniles, only one randomized study has appeared, supporting its efficacy.[6] The drug has a well-established toxicity and side-effect profile. Cyclosporine has been used in the treatment of sight-threatening uveitis for 16 years at this center and we report on its efficacy and tolerability in a large group of patients.

## Materials and Methods

Patients with sight-threatening uveitis are referred to the uvea clinic (UC) at the Eye Hospital. Patients receiving cyclosporine were identified from the clinic database. Cyclosporine was used as first-line or second-line immunosuppression for patients with sight-threatening uveitis, either as a steroid-sparing agent or as sole maintenance for some patients with uveitis and Behçet's disease. Prior to the commencement of treatment, patients were provided with a comprehensive information pamphlet describing drug action, side effects, toxicity, and safety monitoring requirements. Treatment was commenced after informed verbal consent. A management protocol was followed, which required regular monitoring of weight, blood pressure, urinalysis, full blood and differential white cell count, creatinine and electrolytes, liver function, serum lipids, serum magnesium, and trough serum cyclosporine level. When settled on treatment, monitoring occurred at six to eight weekly intervals. Children on cyclosporine were managed jointly with a pediatric rheumatologist.

All patients commencing cyclosporine had treatment objectives which, for those already using oral steroid, included the maintenance of quiescent uveitis or the improvement of active uveitis, with the intention of reducing the steroid dose by 50% or more. For those with Behçet's disease, the objective was to prevent or minimize the frequency of inflammation flare-ups without the use of maintenance steroid treatment. Patients were closely observed during a 3-month trial period, after which a decision was made to continue or to stop treatment.

Following ethical committee approval, data was retrieved from the clinical records of all treated patients including age, sex, diagnosis, treatment course, visual acuity and degree of inflammation before and after treatment, toxicity including abnormal renal function, and hypertension. Patients were also asked to complete a questionnaire on the side effects of treatment. This included direct questions on 14 possible adverse symptoms. Some patients were unavailable for, or failed to respond to a request for the completion of a questionnaire. Thirty-seven completed questionnaires were analyzed.

Intraocular inflammation was assessed before cyclosporine treatment, and after 6 months of treatment. Four parameters of inflammation were recorded as either present or absent: anterior uveitis, vitritis, chorioretinitis, and cystoid macular edema (CME). Vision was assessed as Snellen visual acuity. A change of two lines or more was regarded as a significant change. In addition, visual impairment registrations, effective one-eyed patients (worse than 20/200 in worst eye) and legality for driving, were noted. Cyclosporine effectiveness was judged on the maintenance or improvement of inflammation, on the maintenance or improvement of visual acuity, and on the ability to reduce oral steroid (if used) dosage by 50% or more. For Behçet's disease, the ability to prevent disease attacks on cyclosporine alone was a separate parameter.

## Results

A total of 71 patients attending the UC had received or were receiving cyclosporine. Of these, two were treated for scleritis and 10 had either commenced treatment from other physicians for main causes other than ocular inflammatory disease, or had been treated for uveitis by another ophthalmologist prior to their attendance at the UC. These patients were excluded, leaving a total of 59 (31 female, 28 male) including 5 children under 16, who had initiated treatment with cyclosporine in the UC for the management of sight-threatening uveitis. This was the most frequently used immunosuppressive in the UC. The diagnoses for these 59 patients are given in Table 1, the most common being Behçet's disease-related uveitis.

**Table 1:** Description or diagnosis for patients treated with cyclosporine

Behçet's disease-related uveitis	19
Birdshot retinochoroidopathy	5
Chronic panuveitis, unknown cause	12
Geographic choroiditis	1
HLA-B27-related chronic uveitis	2
Intermediate uveitis, unknown cause	4
Juvenile idiopathic arthritis-related uveitis	4
Primary retinal vasculitis	3
Psoriatic arthropathy-associated uveitis	1
Reiter's disease-related uveitis	1
Sarcoidosis-related chronic panuveitis	2
Sympathetic uveitis	3
Systemic lupus erythematosus-related uveitis	1
Vogt-Koyanagi-Harada syndrome	1
Total	59

Before commencing treatment, patients had been using oral steroid for a mean of 15 months (range 0–48 months) and the mean age at the start of cyclosporine treatment was 37 years (range 6–64 years). The mean starting dose of cyclosporine was 4.2 mg/kg/day and the mean maintenance dose was 3.2 mg/kg/day. The mean duration of treatment (ongoing in 38 patients) was 35 months (range <1–125 months).

Prior to treatment with cyclosporine, 7 patients were active in all four parameters of inflammation (anterior uveitis, vitritis, CME, and chorioretinitis), 19 were active in three parameters, 20 in two, and 11 in one. Two patients were completely quiescent. At the second assessment, all subsets had improved; no patients were active in either four or three parameters, 11 were active in two parameters, 19 in one, and 29 were quiescent. There was clearly an overall substantial shift toward quiescence. Overall 47 patients (78%) improved, 10 were stable (97% improved or stable), and 2 worsened. Anterior chamber activity became quiescent in 25 patients, vitreous activity in 30, CME in 21 and chorioretinitis in 22.

**Figure 1:**
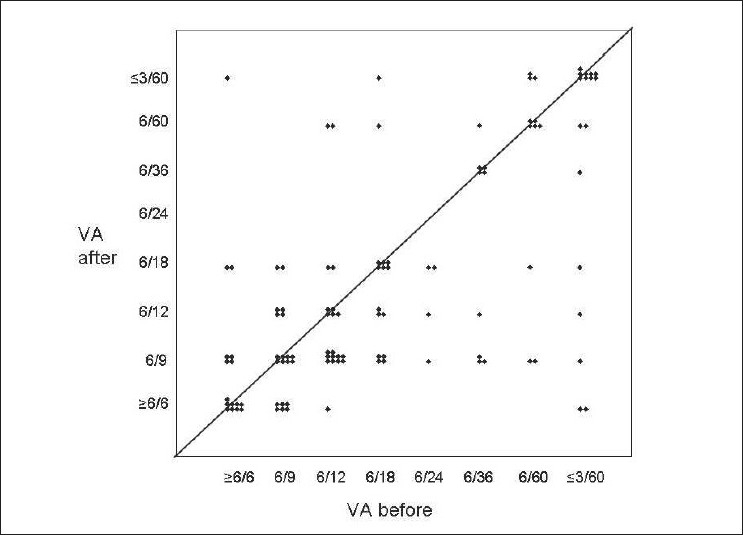
Visual acuity before and after commencing Cyclosporin

Prior to the commencement of cyclosporine treatment, four eyes were blind and another three had been enucleated. Visual acuity change (111 seeing eyes) is shown in Fig. 1. Snellen's visual acuity worsened by two lines or more in the better eye in 5 patients despite treatment, improved by two lines or more in 13, and was stable in the remaining 41. At the latest visit, 6 patients were registered visually impaired, another 15 were effectively one-eyed (acuity in worse eye <20/200), yet 34 (58%) retained acuity adequate for driving (20/30 or better in the better eye). Overall visual acuity was improved or stable in 54 (91%).

Before starting cyclosporine treatment, 49 patients were using prednisolone at a mean dosage of 30.1 mg/day (range 2.5–80 mg/day), but at the second assessment, this had changed to 12.8 mg/day (range 0–80 mg/day), an overall mean reduction of 58%. The target dose reduction of 50% or more was achieved in 25 of the 49 patients (51%), 27 of 49 (55%) reduced dosage to 10 mg/day or less, and 6 had discontinued steroid. Of those 10 patients who were not using steroid prior to cyclosporine, 7 remained without it but 3 required the addition of prednisolone at a mean dosage of 23.3 mg/day (range 10–40 mg/day).

Efficacy was assessed separately for the 19 patients with Behçet's disease. In this subset undergoing a total of 72 patient treatment years (mean 3.8 years), there were a total of eight uveitis flare-ups (three in one patient, one each in five others; mean of one flare-up per nine treatment years). Only one patient lost more than one line of acuity in the better eye, the remainder being improved (4 patients) or stable (14 patients).

Systemic hypertension (blood pressure of 140 or more systolic and/or 95 or more diastolic on two subsequent readings) requiring a dose reduction in cyclosporine (usually in 25% increments) and/or medical treatment occurred in 16 patients (27%). Evidence of reduced renal function shown by a rise in serum creatinine to >110 μmol/l or >30% above baseline level occurred in 18 patients (31%), again requiring dose reduction. There were no cases of malignancy and no cases of severe infection requiring hospital admission. There were no deaths on treatment.

Treatment side effects are shown in Table 2. Only two patients (3%) were entirely free of adverse symptoms. Of the remainder, 86% reported three or more side effects, and 57% reported six or more. The most common adverse symptoms were peripheral burning sensations or paresthesia, followed by fatigue. The treatment was intolerable because of side effects in 12 patients (20%). Adverse symptoms were most common immediately on commencing treatment and it was common for these to improve with time, with or without dose reduction. However, gingivitis and related dental problems, hirsutism, and warts were longer term problems. In contrast, cyclosporine was well tolerated in five children, using a mean maintenance dosage of 3.8 mg/kg/day; none developed hypertension or raised creatinine, and fewer symptomatic side effects were recorded than in adult patients.

**Table 2:** Side effects experienced by patients on cyclosporine treatment

Side effect	% of patients
Paresthesia or burning sensation	70
Fatigue	67
Headache	57
Hirsutism	57
Female patients	62
Male patients	44
Gingivitis, gum swelling	43
Nausea	43
Light-headedness	40
Dyspepsia	40
Tremor	38
Infection	38
General increased tendency	32
Thrush	6
Hospital admissions	0
Palpitations	32
Ankle edema	30
Reduced libido	27
Warts	16

Of the 59 patients using cyclosporine, 37 had also used at least one other immunosuppressive. Eleven had azathioprine added to their regimen (because of the suboptimal efficacy of cyclosporine) and 12 changed to azathioprine. Three had mycophenolate mofetil added to their regime and three changed to mycophenolate. Cyclosporine was the second immunosuppressive in the five children with uveitis, being added to methotrexate (four patients) or substituting for it (one patient). Other replacement immunosuppressives included infliximab (two patients), cyclophosphamide (one patient), and tacrolimus (one patient). At the completion of the study, of 59 patients, 22 (37%) continued to use cyclosporine effectively, with no significant problems; 16 (27%) continued to use it in reduced dosage because of initial side effects (total 64% of patients still on treatment); in 9 patients (15%) it was discontinued because of inadequate efficacy, and in the remaining 12 patients (20%), it was discontinued because of intolerance.

## Discussion

Cyclosporine is an antibiotic, lipophilic cyclic polypeptide synthesized by several fungi including *Trichoderma polysporun* and *Tolypocladium inflatum*. It has a specific action on CD4 lymphocytes, where it inhibits calcineurin, an essential component of the interleukin-2 (IL-2) system. Both IL-2 expression and IL-2 surface receptors are inhibited, which depresses the CD4 lymphocyte's ability to activate and to recruit, so that CD4-driven inflammation is reduced. The drug is metabolized by the hepatic cytochrome P450-A system and is mostly excreted in bile, with 10% excreted in urine.

Our results confirm the efficacy of low-dose (<5 mg/kg/day) treatment in this group, with about 80% of patients demonstrating a reduction in inflammation, about 73% improving or stabilizing vision, and allowing a mean 57% reduction in the oral prednisolone dosage. This has been achieved using a mean maintenance dosage of 3.2 mg/kg/day (range 1.2–5.0), a very low dose regimen allowing low levels of renal toxicity and long-term usage.

In Behçet's disease, cyclosporine has been found to be useful both in reducing the frequency of disease flare-ups and in the maintenance of vision when used in combination with oral steroid[7] or notably, alone,[8] including a long-term successful use. A single-masked comparison of cyclosporine and cyclophosphamide demonstrated the superiority of cyclosporine in Behçet's disease with uveitis.[9] A Cochrane review of pharmacotherapy for Behçet's disease confirmed the protective effect of cyclosporine on eye involvement.[10] Our study strongly supports this; the drug reduces the frequency of uveitis flare-ups to a mean of 1 per 9 treatment years and vision was preserved or improved in 95% of patients over a mean 3.7 years of treatment.

Renal toxicity and hypertension is common and interstitial fibrosis has been shown after 2 years of low-dose (4 mg/kg/day) cyclosporine in a population of uveitis patients,[11] a study which suggests improved safety using a dosage of 3 mg/kg/day or less and reinforces the principle of dose-related nephrotoxicity.

Despite using a mean maintenance dosage as low as 3.2 mg/kg/day, over a quarter of our patients developed systemic hypertension and nearly one-third developed a significant rise in creatinine requiring dose reduction. Twelve patients discontinued the drug because of intolerance and five (9%) because of hypertension and/or depressed renal function. There is no completely safe dose for cyclosporine in relation to renal function, but toxicity is dose related and using a very low dose regimen, we have minimized problems in our patient group.

Migraine-type headache occurs frequently in cyclosporine usage.[12] Our study revealed a headache rate of 57%, though this reduced substantially with time and dose reduction. Focal neurotoxicity may occur, and it has been suggested[13] that cyclosporine may actually induce neurological involvement in Behçet's disease. Our study could not confirm this, revealing one episode of acute neuro-Behçet (1/19, 5%) manifesting as stroke, in a patient on cyclosporine.

There is a small risk of the development of malignancy on cyclosporine treatment, especially lymphoma and carcinoma of the skin and mucosae. This risk appears to be smaller for those with uveitis than those with multisystem inflammatory disease or organ transplant using the same drug. No episodes of malignancy have occurred in our patients, but in recognition of this risk, we have introduced into our clinic a proactive nurse-led system to encourage self-examination and reporting for skin lesions.

Although completed by only a representative sample of patients, our results show a high incidence of side effects [Table 2], with peripheral burning/discomfort, fatigue, hirsutism, and headache being the most problematic. Seven patients (12%) discontinued treatment because these problems were intolerable.

Alternatives to systemic cyclosporine have been sought. Tacrolimus is well established and a better safety profile is claimed.[14] Intraocular drug delivery[15] is the subject of research, as are alternative calcineurin inhibitors. However, cyclosporine currently remains the most-used drug in this group.

In conclusion, we have reported the largest series to date, of patients using oral low-dose cyclosporine in the management of sight-threatening uveitis, totaling 153 patient/years of treatment. The drug has proven to be of great value in maintaining vision and reducing inflammation, with a manageable toxicity profile. It is safe for long-term usage, with careful monitoring. It is the first-choice immunosuppressive in this clinic. We recommend adherence to a strict management protocol, including regular formal assessment of patient tolerance.

## References

[1] Borel JF, White DJ (1982). The history of cyclosporin A and its significance. Cyclosporin A; Proceedings of an International Conference on Cylosporin A, Cambridge, September 1981.

[2] Ruegger A, Kuhn M, Lichti H, Loosli HR, Huguenin R, Quiquerez C (1976). Cyclosporin A, a peptide metabolite from Trichoderma polysporum Rifai, with a remarkable immunosuppressive activity. Helv Chim Acta.

[3] Coster DJ, Shepherd WF, Fook TC, Rice NS, Jones BR (1979). Prolonged survival of corneal allografts in rabbits treated with cyclosporin A. Lancet.

[4] Nussenblatt RB, Rodrigues MM, Salinas-Carmona M, Gery I, Cevario S, Wacker W (1982). Modulation of experimental autommune uveitis with cyclosporin A. Arch Ophthalmol.

[5] Nussenblatt RB, Palestine AG, Rook AH, Scher I (1983). Treatment of intraocular inflammatory disease with cylosporin A. Lancet.

[6] De Vries J, Baarsma GS, Zaal MJ, Boen-Tan TN, Rothova A, Buitenhuis HJ (1990). Cyclosporin in the treatment of severe chronic idiopathic uveitis. Br J Ophthalmol.

[7] Whitcup SM, Salvo EC, Nussenblatt RB (1994). Combined cyclosporine and corticosteroid therapy for sight-threatening uveitis in Behcet's disease. Am J Ophthalmol.

[8] Diaz-Llopis M, Cervera M, Menezo JL (1990). Cyclosporin A treatment of Behcet's disease: a long-term study. Curr Eye Res.

[9] Ozyazgan Y, Yurdakul S, Yazici H, Tuzun B, Iscimen A, Tuzun Y (1992). Low dose cyclosporin A versus pulsed cyclophosphamide in Behcet's syndrome: a single masked trial. Br J Ophthalmol.

[10] Saenz A, Ausejo M, Shea B, Wells G, Welch V, Tugwell P (2000). Pharmacotherapy for Behcet's syndrome. Cochrane Database Syst Rev.

[11] Bagnis CI, du Montcel ST, Beaufils H, Jouanneau C, Jaudon MC, Maksud P (2002). Long-term renal effects of low-dose cyclosporine in uveitis-treated patients: follow-up study. J Am Soc Nephrol.

[12] Ferrari U, Empi M, Kim KS, Sostak P, Forderreuther S, Straube A (2005). Calcineurin inhibitor-induced headache: clinical characteristics and possible mechanisms. Headache.

[13] Akman-Demir G, Ayranci O, Kurtuncu M, Vanli EN, Mutlu M, Tugal-Tutkun I (2008). Cyclosporine for Behcet's uveitis: is it associated with an increased risk of neurological involvement?. Clin Exp Rheumatol.

[14] Murphy CC, Greiner K, Piskova J, Duncan L, Frost NA, Forrester JV (2005). Cyclosporine vs tacrolimus therapy for posterior and intermediate uveitis. Arch Ophthalmol.

[15] Dong X, Shi W, Yuan G, Xie L, Wang S, Lin P (2006). Intravitreal implantation of the biodegradable cyclosporin. A drug delivery system for experimental chronic uveitis. Graefes Arch Clin Exp Ophthalmol.

